# Strategy for Fault Diagnosis on Train Plug Doors Using Audio Sensors †

**DOI:** 10.3390/s19010003

**Published:** 2018-12-20

**Authors:** Yongkui Sun, Guo Xie, Yuan Cao, Tao Wen

**Affiliations:** 1 School of Electronic and Information Engineering, Beijing Jiaotong University, Beijing 100044, China; 17111042@bjtu.edu.cn (Y.S.); t.wen@bjtu.edu.cn (T.W.); 2 Shaanxi Key Laboratory of Complex System Control and Intelligent Information Processing, Xi’an University of Technology, Xi’an 710048, China; 3 National Engineering Research Center of Rail Transportation Operation and Control System, Beijing Jiaotong University, Beijing 100044, China

**Keywords:** fault diagnosis, train plug doors, multi-scale permutation entropy (MNPE), improved particle swarm optimization (IPSO), multi-class SVM

## Abstract

As the only entry/exit for passengers getting on and off a train, the train plug door is of great importance to keep train operation safe and reliable. As signal processing technologies develop rapidly, taking the easy acquisition advantages of sound signals, a novel fault diagnosis method for train plug doors using multi-scale normalized permutation entropy (MNPE) and an improved particle swarm optimization based multi-class support vector machine (IPSO-MSVM) is proposed. Firstly, sound samples are collected using high-precision audio sensor. In the features extraction process, a hybrid method blending empirical mode decomposition (EMD), multi-scale permutation entropy (MNPE) with Fisher discrimination criterion is utilized. First, EMD is used to decompose each sound signal into several intrinsic mode functions (IMFs) and a residue for stationary processing. Then, MNPE features are extracted from the IMFs. To obtain the most significant features, the Fisher discrimination criterion is further applied. To address the time-consuming defects of traditional grid based method for selecting the optimal parameters of multi-class SVM, an improved PSO (IPSO) is proposed. The superiority of the IPSO-MSVM model and the hybrid feature extraction method was tested on the collected sound samples by comparing to commonly applied methods. Results indicate the identification accuracy of the proposed method is highest, which reaches 90.54%, demonstrating its feasibility.

## 1. Introduction

Fault diagnosis plays an important role in various fields [1,2,3,4]. In recent years, high-speed train technology has been developing rapidly. Some works on it have been done, such as fuzzy predictive control technology in automatic train operation [5] and fault diagnosis for railway signal system using local fractional method [6]. The high-speed train is a very complex system that contains many devices. Condition monitoring of these devices is essential to keep the train operating in a safe and reliable state. The train plug door is an important component for passengers getting on and off the train. However, as the train plug door opens and closes in a high-frequency condition, some faults appear. At present, train plug door maintenance methods include time based maintenance (TBM) and manual troubleshooting. To reduce the cost of manpower and material resources, an efficient and automatic fault diagnosis method for train plug doors needs to be developed.

In recent years, research regarding fault diagnosis of train plug doors has been conducted focusing on statistical analysis methods, expert system, and model based methods. Fault tree analysis (FTA) is applied to fault diagnosis of train plug doors, and the importance of each component towards a particular failure mode is obtained for supporting maintenance [7]. However, statistical analysis methods belong to post-analysis methods requiring many historical data. Due to the limitations of their attributes, they cannot identify a certain fault type. Failure mode and effects analysis (FMEA) is used to evaluate the reliability of high-speed train door system [8]. Bayesian network model using expert system knowledge is developed to diagnose the faults of sliding plug doors [9]. A remote monitoring system for metro door system is developed, which automatically sends the collected data to remote engineers or experts for analysis [10]. However, the effectiveness of the expert knowledge based analysis is biased by subjective experiences. Model based methods are proposed by establishing a mathematical model of train doors, and a parameter is set to reflect the working condition [11,12,13]. However, the accuracy parameters in the established model are difficult to obtain in practice. Besides, an accurate model is difficult to develop as the train door system is a complex system. Data-driven methods can provide the possibility to address the above defects. We developed a method based on sound recognition using a two-layer decomposition feature extraction method (EMD-WPD) combining EMD with wavelet package decomposition (WPD) and multi-class SVM [14]. In this paper, one more fault type is collected. The previously proposed method performs poorly on the newly added type. Hence, a novel efficient sound based strategy for automatic fault diagnosis of train plug doors is proposed.

It is noted that the sensor is a preferable way to acquire useful data in life and industry activities due to its convenience and good performance. In recent years, sensors are widely used in various fields. Especially, some applications in the field of fault diagnosis are investigated, such as bearings [15,16] and railway point machines [17]. Moreover, there are some advantages for fault diagnosis via sound signals. For example, sound signals are easy to acquire using audio sensors. In addition, sound signals collection does not cause interference to the original electric equipment. As signal processing technologies develop rapidly, some advantageous technologies are proposed, such as wavelet transform [18] and EMD [19]. Especially, EMD is an adaptive and data-driven method to decompose a signal into a series of IMFs and a residue, which is suitable for nonlinear and non-stationary signals analysis. Although there are some improved EMD versions, such as ensemble EMD [20] and complete ensemble EMD [21], they are very time-consuming. Thus, EMD is utilized to do stationary processing of the original sound signals.

In the fault diagnosis process, one of the most important steps is fault feature extraction. Effective fault features are the basis of high identification accuracy. Permutation entropy (PE) was proposed in 2002 [22]. PE reflects the numerical relationship of neighboring values. Due to its simplicity, robustness and fast calculation, it is used in various fields, such as revealing abnormalities of cerebral activity by analyzing electroencephalograph (EEG) signals [23,24] and fault diagnosis for roller bearings [25]. Since the sound signals obtained from mechanical systems are complex, PE may be inefficient. To deal with this problem, multi-scale permutation entropy (MPE) is introduced to extract fault features [26], which captures more information from time series data. Considering the accuracy of fault diagnosis on train plug doors, MPE is used in method proposed in this paper. Factor scale [27] is an important parameter related to the quality of MPE features, which should be selected properly.

A classification technique is required to provide fault diagnosis. SVM is a powerful classification tool based on structure risk minimization principle with strong generation ability [28]. Compared to artificial neural networks (ANN), it is more competitive when only limited data are available, thus is widely used in classification tasks [29,30]. Two hyperparameters [31] should be selected properly in SVM. The most traditional method to tune the hyperparameters is called grid search [32], but this method is very time-consuming [33]. Particle swarm optimization (PSO) is a more efficient method to search the optimal solution using few parameters [34]. Hence, PSO is selected in the scope of this paper to tune the SVM hyperparameters.

In this paper, a novel sound based fault diagnosis strategy for train plug doors is proposed. The effective fault features are extracted using a hybrid method blending EMD and MNPE with the Fisher discrimination criterion. First, EMD is used to decompose the sound signals for stationary processing. Subsequently, MNPE features of each IMF are obtained by applying feature extraction procedures. Further, the Fisher discrimination criterion is used to select the optimal discrimination features to improve the accuracy of the fault diagnosis. In addition, the relationship between scale factor and identification accuracy is discussed to determine the optimal scale factor. Apart from fault features, the performance of the classifier also contributes to the fault identification accuracy. To optimize the multi-class SVM, an improved PSO algorithm is introduced. Finally, the multi-class SVM model is trained and verified using training set and test set, respectively.

The reminder of this paper is organized as follows. Section 2 introduces the data and the whole diagnosis framework. Section 3 introduces the hybrid feature extraction method combining EMD, MNPE and the Fisher discrimination criterion. In Section 4, IPSO-MSVM is described. Experimental results are shown in Section 5. In addition, the results are analyzed and discussed. Section 6 concludes the paper and lists future work.

## 2. Experimental Setup

### 2.1. Data Acquisition

**Figure 1:**
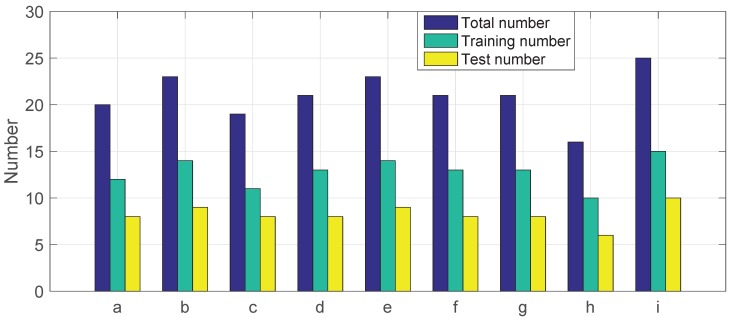
Numbers of recorded sound signals from Type-a to Type-i.

The sound data for this study were obtained from Hankou Multiple Unit Operation Depot, Wuhan Railway Bureau. The reviewed high-speed train model is the China Railway CRH5 Size A (CRH5A). A portable recorder PCM-D50 was used to collect the sound signals of train plug doors with a sampling rate of 44.1 kHz. Nine types and 189 sets of sound samples were collected. In this paper, 60% of the data were used as training set. The numbers of training samples and test samples of each type are shown in Figure 1.

**Figure 2:**
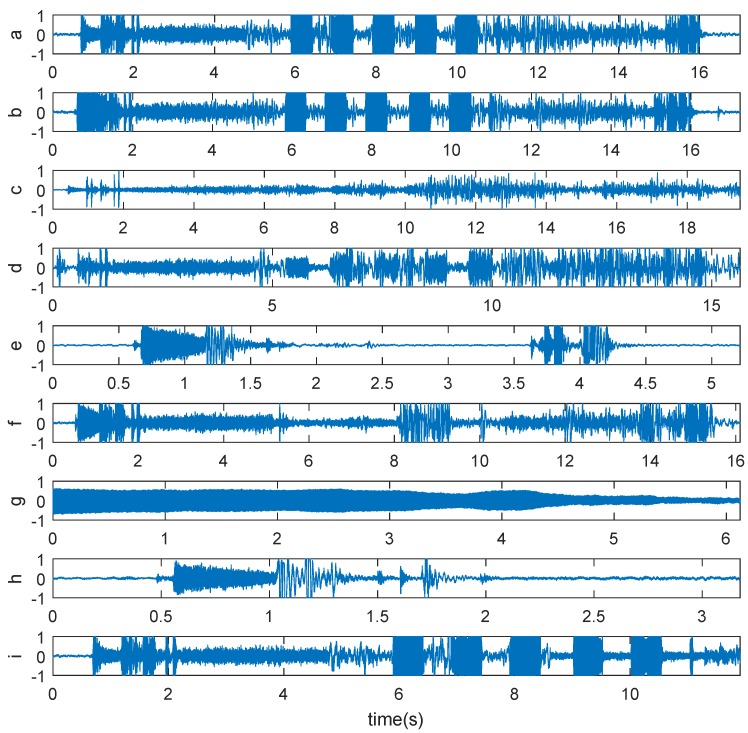
Time-domain waveforms of sound signals from Type-a to Type-i.

Type a–i represent the types of sound signals corresponding to normal, silencing valve missing, 98% limit switch failure, pneumatic lock failure, unlocking motor failure, buzzer failure, urgent open alarming, drive motor failure, and the door cannot close because of obstacles, respectively. The detailed descriptions about Type-a to Type-h can be seen in our previous work [14]. In the case of Type-i, the door will open when it encounters an obstacle in the process of closing. Thus, door panel impact sound and locking sound of pneumatic lock do not exist. The respective time-domain waveforms are given in Figure 2. It can be seen that some types behave very similarly in the time-domain, such as Type-a and Type-b. Therefore, an efficient feature extraction method needs to be developed.

### 2.2. Intelligent Diagnosis Strategy for Train Plug Doors

**Figure 3:**
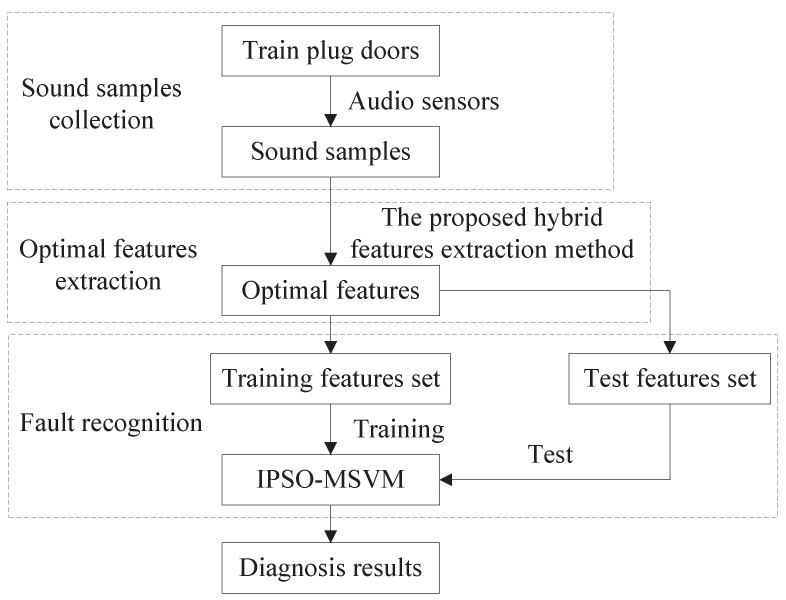
The proposed novel intelligent fault diagnosis method for train plug doors.

On the basis of the proposed hybrid feature extraction method and the IPOS-MSVM model, a novel intelligent fault diagnosis method based on sound signals is proposed to identify different working conditions of train plug doors, which is shown in Figure 3 and includes the following steps:

- Step1: Sound samples collection
  To ensure the data reliability and fault identification accuracy, collect sound samples under different working conditions using high-precision audio sensor.
- Step2: Optimal features extraction
  Select proper parameters for the proposed hybrid features extraction method. Then, obtain the optimal features using the hybrid method.
- Step3: Fault recognition using IPSO-MSVM
  Divide the extracted features into training set and test set by split ratio of 6:4. The training set is used to train the classifier, whereas the test set is used to verify the effectiveness of the proposed diagnosis method.

## 3. Features Extraction Methods for Sound Signals

### 3.1. Empirical Mode Decomposition

EMD is a data-driven signal decomposition method, especially suited for non-stationary and nonlinear signals. The IMFs obtained via EMD are relatively stationary and capture a certain frequency bandwidth. According to the definitions proposed by Huang, each IMF should meet the following two conditions [35]: (a) over the entire time range, the number of extrema and zero crossings must be equal or differ at most by one; and (b) the mean value of upper and lower envelopes should be zero at any point.

The procedures of EMD can be summarized as follows.

(1) Find all of the extrema of signal x(t).

(2) Obtain the upper envelope $e_{u}\left(t\right)$ and lower envelope $e_{l}\left(t\right)$ by applying cubic spline interpolation to the local maximum and local minimum, respectively.

(3) Calculate the average of $e_{u}\left(t\right)$ and $e_{l}\left(t\right)$ as

(1) $$\begin{array}{c} m\left(t\right)=\frac{e_{u}\left(t\right)+e_{l}\left(t\right)}{2} \end{array}$$

(4) Obtain h(t) using

(2) $$\begin{array}{c} h(t)=x(t)-m(t) \end{array}$$

(5) Evaluate: If h(t) satisfies the two conditions, an IMF is obtained imf(t)=h(t); otherwise, repeat Steps (1)–(5) until the standard deviation (SD) is smaller than a certain value.

(3) $$\begin{array}{c} SD=\sum_{i=1}^{N}\frac{|h_{k}\left(t\right)-h_{k-1}{\left(t\right)|}^{2}}{h_{k-1}^{2}\left(t\right)} \end{array}$$

where $h_{k-1}\left(t\right)$ and $h_{k}\left(t\right)$ are the results of the (k-1)th and the *k*th iteration. SD is typically set between 0.2 and 0.3.

When the first IMF is obtained as $imf_{1}\left(t\right)=h\left(t\right)$, the residual signal can be obtained using $r_{1}\left(t\right)=x\left(t\right)-imf_{1}\left(t\right)$. By applying Steps (1)–(5) to $r_{1}\left(t\right)$, the second IMF can be obtained. The iteration process continues until the extrema number of the residual signal is less than two.

Finally, the the original signal x(t) can be decomposed into several IMFs and a residue, as

(4) $$\begin{array}{c} x\left(t\right)=\sum_{i=1}^{n}imf_{i}\left(t\right)+r\left(t\right) \end{array}$$

where $imf_{i}\left(t\right)$ is the *i*th IMF, r(t) is the residue, and *n* is the number of IMFs.

### 3.2. Multi-Scale Normalized Permutation Entropy

In 1980, Packard et al. proposed a method for phase space reconstruction of time series, named coordinate delay reconstruction method [36]. The basic principle of this method is that a time-delay reconstruction technique is used to reconstruct a phase space, which is topologically isomorphic with the original time series. In other words, the phase space reconstruction method can transform a time series into high-dimensional series named phase space. For a time series x(i) of length *N*, the reconstructed phase space can be expressed as follows.

(5) $$\left\{\begin{array}{c} X(1)=x(1),x(1+\tau ),\cdots ,x(1+(m-1)\tau )\\ \vdots \\ X(i)=x(i),x(i+\tau ),\cdots ,x(i+(m-1)\tau )\\ \vdots \\ X(N-(m-1)\tau )=x(N-(m-1)\tau ),x(N-(m-2)\tau ),\cdots ,x(N) \end{array}\right.$$

where *m* is the embedding dimension and τ is the time lag. Considering the numerical relationship, X(i) can be arranged in ascending order as

(6) $$\begin{array}{c} x\left(i+r_{1}\tau \right)\leq x\left(i+r_{2}\tau \right)\leq \cdots \leq x\left(i+r_{m}\tau \right) \end{array}$$

where $r_{k}$ is an integer satisfying $0\leq r_{k}\leq m-1$ and $r_{i}\neq r_{j}$. Then, the vector $\left(r_{1},r_{2},\cdots ,r_{m}\right)$ can be defined as a permutation.

It is easy to note that there are total m! possible permutations for each X(i),i=1,2,⋯,N. The statistical numbers $\left(N_{1},N_{2},\cdots ,N_{m!}\right)$ of each permutation are obtained. Then, the PE of *m*-order can be obtained as

(7) $$\begin{array}{c} H_{PE}=-\sum_{i=1}^{m!}p_{i}\ln\left(p_{i}\right),p_{i}=N_{i}/\sum_{i=1}^{m!}N_{i} \end{array}$$

where $p_{i}$ represents the probability of the *i*th permutation. The maximum value of $H_{PE}$ is ln(m!) when the probabilities of all permutations are equal. Then, the normalized PE (NPE) can be obtained by

(8) $$\begin{array}{c} H_{NPE}=H_{PE}/\ln\left(m!\right) \end{array}$$

where $0\leq H_{NPE}\leq 1$. NPE can reflect the degree of randomness of time series to some extent. A larger $H_{NPE}$ indicates the time series is more random [37].

The NPE has the disadvantage of neglecting the values of the time series. The MNPE can address the disadvantage. The MNPE includes more useful information about a nonlinear and complex system. The main idea of MNPE extraction procedure is to construct the coarse-grained time series by calculating the average of data inside non-overlapping windows of length *l* (scale factor), which takes the series values into consideration. The coarse-grained process is realized by

(9) $$\begin{array}{c} y^{l}\left(i\right)=\frac{1}{l}\sum_{j=(i-1)l+1}^{il}x\left(j\right),i=1,2,\cdots ,N/l \end{array}$$

where $y^{l}$ is the obtained coarse-grained time series. When the length of non-overlapping window is 1, the extracted NPE feature matches the one of the original time series. By selecting scale factor within a certain range, a series of NPE features can be obtained and combined together as MNPE features. As the values of the time series are considered, MNPE can contain more valuable information.

### 3.3. The Hybrid Feature Extraction Method

**Figure 4:**
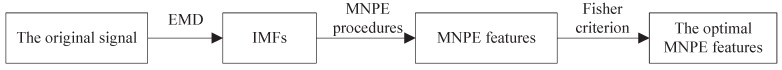
The procedures of the hybrid feature extraction method.

It is noted that the collected sound signals may contain some noise, which may result in low performance for fault diagnosis. To obtain the most effective fault features and reduce the influences from noise, a hybrid method combining EMD, MNPE, and Fisher discrimination criterion [38] is proposed. The procedures are given in Figure 4.

First, EMD is applied to decomposed the original sound signal into several IMFs. Then, MNPE features of the IMFs are obtained. Finally, the optimal MNPE features are obtained by using Fisher discrimination criterion. A larger discrimination function value *J* indicates the corresponding features have more obvious discrimination effects.

## 4. Multi-Class SVM Based on Improved PSO

### 4.1. Multi-Class SVM

**Figure 5:**
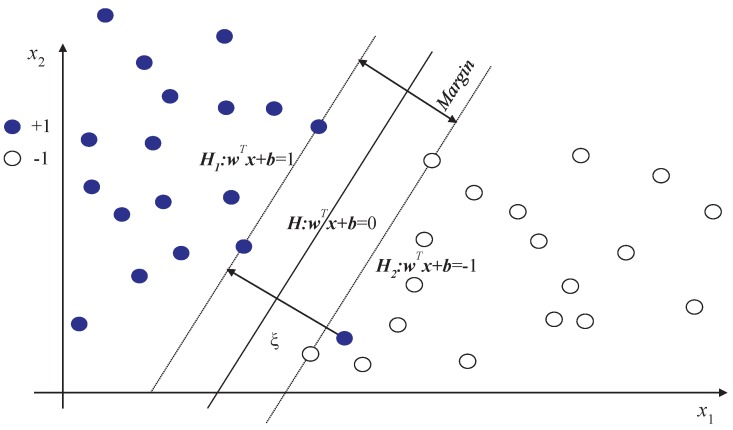
The principle of SVM.

SVM is a supervised learning algorithm, which is the preferable method for small set clustering. It performs well in classification of limited data by adopting the structural risk minimization principle. Initially, SVM is used to solve a two-class problem. Taking a two-dimensional problem as an example, the main idea of SVM is to find the optimal hyperplane with maximum margin for linear classification problems, as shown in Figure 5.

According to the idea of SVM, the classification problem can be transformed to a convex optimization problem as follows:

(10) $$\begin{array}{c} min\;\frac{1}{2}w^{T}w+C\sum_{i=1}^{\beta }\xi_{i}\\ s.t.\;y_{i}\left(w^{T}x_{i}+b\right)\geq 1-\xi_{i}\\ \;\;\xi_{i}\geq 0 \end{array}$$

where $\xi_{i}$ is the penalty for misclassification samples; *C* is a positive constant called regularization parameter; and *H* is the optimal hyperplane. Given a sample $x^{\prime }$, the prediction label can be obtained using $sgn(w^{T}x^{\prime }+b)$.

SVM can also be used to solve nonlinear problems by introducing the kernel function. The kernel function is used to map the inseparable samples to higher even infinite dimension space where these samples become separable. In addition, the introduction of a kernel function can solve the problem of dimension disaster. The kernel function is defined as $K\left(x_{i},x_{j}\right)=\phi {\left(x_{i}\right)}^{T}\phi {\left(x_{j}\right)}^{T}$. A commonly used kernel function is the radial basis function (RBF) expressed as follows:

(11) $$\begin{array}{c} K\left(x_{i},x_{j}\right)=\exp(-g||x_{i}-x_{j}{\left|\right|}^{2}),g>0 \end{array}$$

where *g* is a kernel parameter.

SVM can be extended to solve multi-class classification problems. Multi-class SVM includes two modes: one versus one (OVO SVMs) [39] and one versus rest (OVR SVMs). Although fewer SVMs are required, OVR SVMs comes with the disadvantages of low training speed and sample asymmetry. In addition, if a new class is included, all SVMs have to be retrained. If the number of classes is not very large, OVO SVMs can solve the problems of OVR SVMs. Therefore, OVO SVMs is used in this paper. The main idea of OVO SVMs is to establish a SVM between each pair of classes. Therefore, c(c-1)/2 SVMs need to be constructed, where *c* is the number of classes. The class of a given sample can be predicted by the highest vote of all SVMs.

According to Equations (9) and (10), two parameters (*C* and *g*) need to be determined. To obtain the most optimal parameters, *k*-fold cross-validation method is used. In *k*-fold cross-validation, the training set is divided into *k* subsets of equal size. k-1 subsets are used to train multi-class SVM model, while the remaining one is used to test the performance of the model. The parameters can be determined using a measure of cross-validation accuracy.

### 4.2. Improved PSO

Optimization methods have been applied in various areas of research [40]. PSO is a global optimization algorithm, the main idea of which originates from the studies on predation behavior of birds. Due to the advantages of simplicity and fewer parameters, PSO is widely used. In PSO algorithm, *M* particles fly in the *d*-dimensional search space to search for the optimal solution by iteration process. In the *k*th iteration, the *i*th particle has the current position $x_{i}^{k}=\left(x_{i1}^{k},x_{i2}^{k},\cdots ,x_{id}^{k}\right)$ with the current velocity $v_{i}^{k}=\left(v_{i1}^{k},v_{i2}^{k},\cdots ,v_{id}^{k}\right)$. Each particle memorizes its optimal position $p_{i}^{k}=\left(p_{i1}^{k},p_{i2}^{k},\cdots ,p_{id}^{k}\right)$ in historical iteration process. The optimal position of all particles is $p_{g}^{k}=\left(p_{g1}^{k},p_{g2}^{k},\cdots ,p_{gd}^{k}\right)$. Then, the position and velocity of the particles can be updated as follows:

(12) $$\begin{array}{c} v_{ij}^{k+1}=wv_{ij}^{k}+c_{1}\xi \left(p_{ij}^{k}-x_{ij}^{k}\right)+c_{2}\eta \left(p_{pj}^{k}-x_{gj}^{k}\right) \end{array}$$

(13) $$\begin{array}{c} x_{ij}^{k+1}=x_{ij}^{k}+v_{ij}^{k+1} \end{array}$$

where *w* is the inertia weight factor; $c_{1}$ and $c_{2}$ are the acceleration coefficients; and ξ and η are two independent random numbers satisfying uniform distribution in the range [0, 1].

Some studies indicate that the inertia weight factor *w* is a key parameter of PSO [41]. The larger *w* is, the stronger the global search ability is, but with the disadvantage of low local search ability. A relatively small *w* makes PSO be with strong local search ability but the obtained solution may be not the global optimal one. Thus, the trade-off between global and local search ability is considered. An improved PSO (IPSO) based on decrement inertia weight factor strategy is adopted as:

(14) $$\begin{array}{c} w=w_{end}{\left(w_{start}/w_{end}\right)}^{1/(1+\alpha i/t_{max})} \end{array}$$

where $w_{start}$ and $w_{end}$ are the maximum and minimum of inertia weight factor, respectively; $t_{max}$ is the maximum of the iteration; and α is a positive constant to control the decreasing velocity of *w*.

In the early iteration stage, the larger *w* makes the particle swarm reach the global optimal region quickly. Then, the decreasing *w* makes the particle swarm get more and more accurate solutions in the region.

### 4.3. Multi-Class SVM Optimized via IPSO

**Figure 6:**
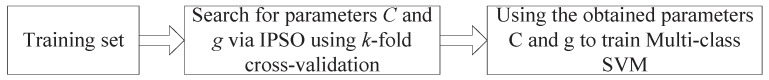
The flow of IPSO-MSVM.

According to the above introduction of multi-class SVM, two parameters (*C* and *g*) need to be determined. Because the two parameters have a vital influence on the classification accuracy, they need to be selected properly. Taking the strong global search ability, IPSO based multi-class SVM (IPSO-MSVM) is proposed. The flow of IPSO-MSVM is given in Figure 6.

After the optimal parameters *C* and *g* are obtained by IPSO using *k*-fold cross-validation, they are used to train the multi-class SVM model for classification.

## 5. Results and Discussions

### 5.1. The Selection of Optimal Scale Factor Range

**Figure 7:**
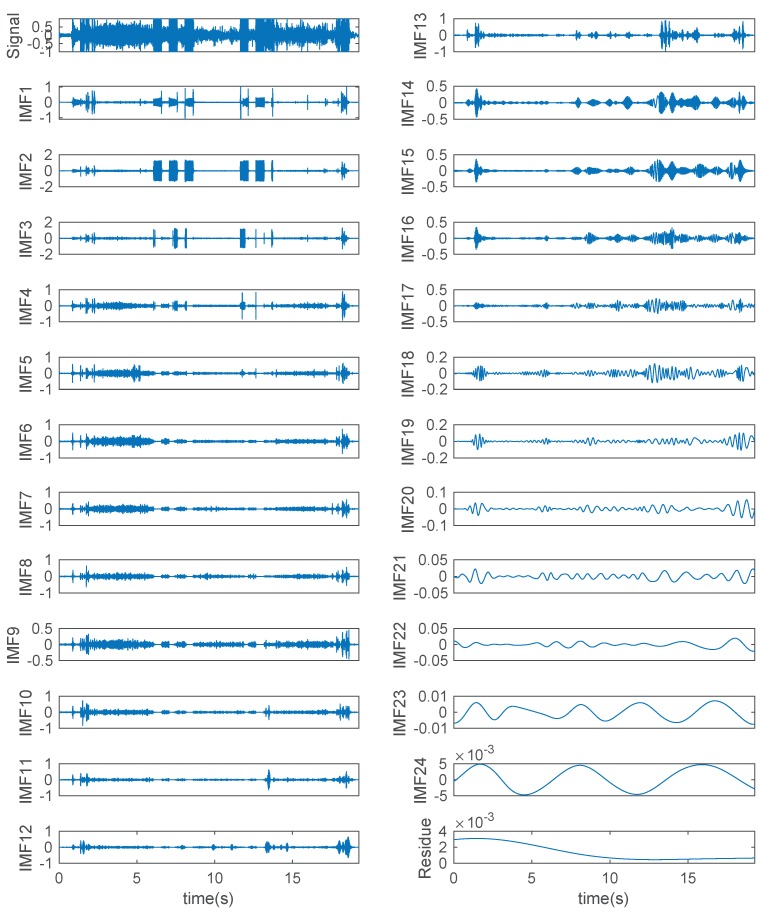
EMD results of a sound signal of Type-a.

Taking a sound signal of Type-a as an example, the decomposition results via EMD are shown in Figure 7. It can be seen that the original signal was decomposed into 24 IMFs and a residue. As the iterative decomposition continued, the frequency of IMF became lower and lower. The residue represents the changing trend of the original signal. The anterior IMFs contain high-frequency useful information, whereas the last several IMFs are with lower amplitude and frequency. By analyzing the EMD results of all types of sound signals, it was found that the number of IMFs ranged from 13 to 30. Therefore, the first 13 IMFs were selected as the research objects.

In the MNPE features extraction process, *m* is a key parameter. A small *m* results in too few permutations, which will not allow extracting more useful information from the signal. In contrast, if *m* is too large, the calculation cost will be expensive. In this study, *m* was selected as 4, and τ was set as 1. By applying MNPE features extraction procedures to the first 13 IMFs, a feature matrix for each sound signal could be obtained. To obtain the most significant discrimination feature and improve the efficiency and identification accuracy of IPSO-MSVM, Fisher discrimination criterion was applied. Then, the selected MNPE features were integrated into a feature vector for classification analysis.

**Figure 8:**
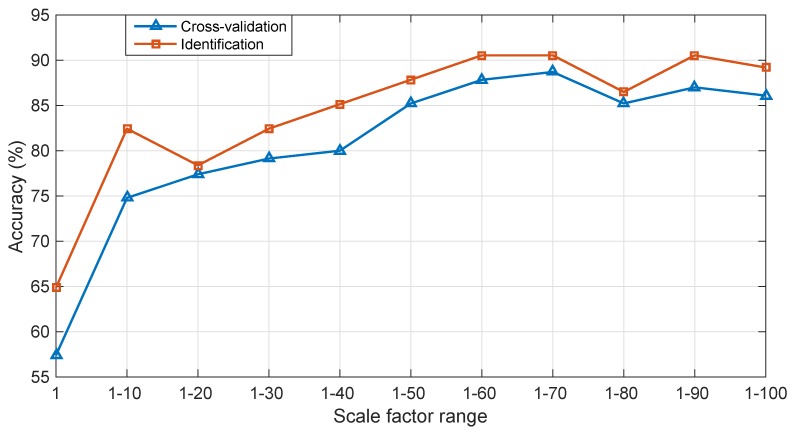
Cross-validation accuracy and identification accuracy using IPSO-MSVM under different scale factor ranges.

Moreover, the range of scale factor also plays an important role in fault diagnosis performance. The maximum length *l* of the non-overlapping window in coarse-grained process was set as 100. To determine the optimal scale factor range, the obtained optimal feature vectors were used to train IPSO-MSVM for verification. The parameters used in this study were set as $w_{start}=0.9$, $w_{end}=0.15$, α=10, $t_{max}=400$, $c_{1}=1.7$, and $c_{2}=1.7$, and three-fold cross validation was adopted. The search ranges of *C* and *g* were both set as $\left[10^{-2}:10^{3}\right]$. The best cross-validation accuracy and identification accuracy using IPSO-MSVM under 11 groups of scale factor ranges are shown in Figure 8.

It could be concluded from the results in Figure 8 that IPSO-MSVM model trained using MNPE features performs better than the one trained using NPE features (when scale factor range is 1). In addition, when scale factor is smaller than 70, the best cross-validation accuracy becomes higher and higher as the scale factor range increases. When the scale factor range continues to increase, the best cross-validation accuracy becomes a little lower than the one when scale factor range is 1–70. Therefore, the scale factor range was selected as 1–70.

### 5.2. Diagnosis Results Comparison among BP Neural Network, 1NN, PSO-MSVM, and IPSO-MSVM Classifiers

**Figure 9:**
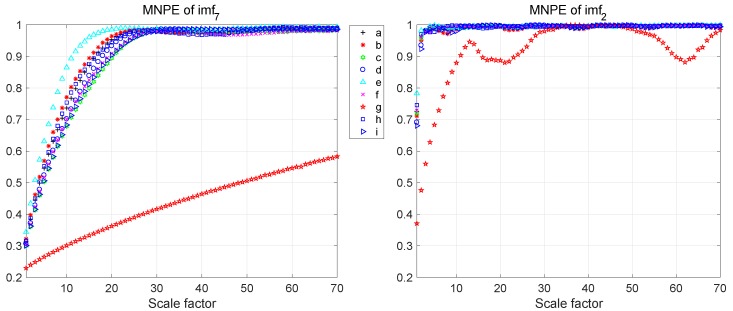
MNPE features comparison of $imf_{7}$ and $imf_{2}$.

To select the most significant features, the Fisher discrimination criterion was utilized. The obtained discrimination function values (*J*) via the Fisher discrimination criterion when scale factor range is 1–70 are shown in Table 1. According to the discrimination function values, it was found that the discrimination function values of $imf_{6}$, $imf_{7}$ and $imf_{8}$ are obviously larger than the others. Thus, the MNPE features of $imf_{6}$, $imf_{7}$ and $imf_{8}$ were selected, and integrated into the feature vector. To intuitively show the superiority of the selected features, the MNPE features of $imf_{7}$ and $imf_{2}$ are shown in Figure 9. It can be seen that the MNPE features of $imf_{2}$ are almost inseparatable except Type-g, whereas they are more separatable in $imf_{7}$. In addition, as the scale factor increases, the NPE becomes larger and larger in overall trend. After the scale factor reaches a certain value, the NPE fluctuates in a small range.

**Figure 10:**
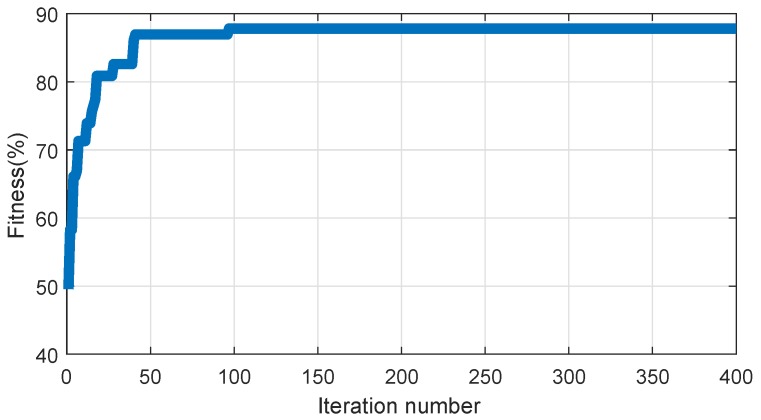
The results of IPSO.

Then, the obtained feature vectors of the training set were used to train the IPSO-MSVM. By using IPSO, the optimal parameters for multi-class SVM were obtained as C=478.3604 and g=0.9163. The relation between fitness values and the iteration number is shown in Figure 10. It can be seen that IPSO reaches the optimal solution when the iteration number is about 100.

**Figure 11:**
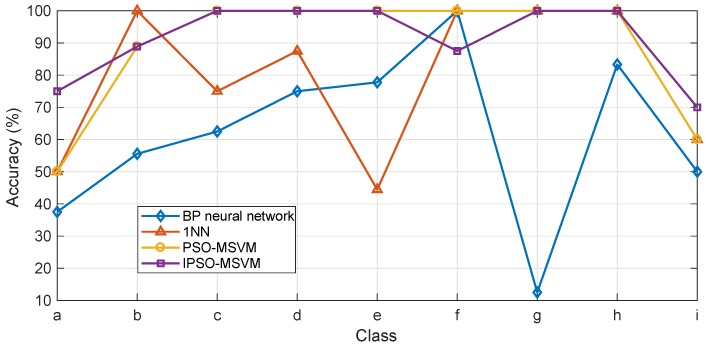
The identification accuracy of each class using different classifiers.

Finally, the obtained optimal parameters were used to train the multi-class SVM. Backpropagation (BP) neural network classifier, 1 Nearest Neighbor (1NN) classifier, and multi-class SVM based on PSO with fixed inertia weight (PSO-MSVM) were used to demonstrate the effectiveness and superiority of the selected approach. The identification accuracy of each class and the identification results are shown in Figure 11 and Table 2, respectively.

In Figure 11, it can be intuitively seen that the overall identification accuracy using IPSO-MSVM is higher compared to other commonly used classifiers. The BP neural network classifier performs worst due to small sample set. In addition, the identification accuracy on Type-a and Type-g is even lower than 50%, demonstrating it is not a suitable approach if only small datasets are available. Overall, 1NN classifier performs better compared to the BP neural network classifier. It can be seen in Table 2 that PSO-MSVM can further improve the overall identification accuracy compared to both the BP neural network classifier and 1NN classifier. However, the identification accuracy on Type-a and Type-i is relatively low. Note that the overall identification accuracy using IPSO-MSVM reaches 90.54%, which is the highest. Meanwhile, the identification accuracy on each class is also higher than the others except a little lower on Type-b and Type-f, indicating that the proposed IPSO-MSVM model performs best on fault diagnosis for train plug doors.

### 5.3. Diagnosis Results Comparison among Different Feature Extraction Methods

**Figure 12:**
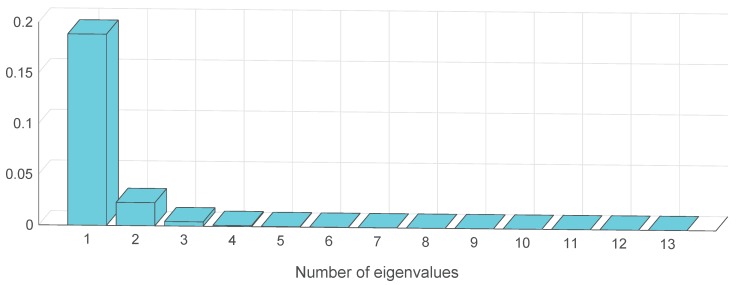
Eigenvalues by applying PCA to 13-dimensional MNPE features.

To verify the effectiveness and superiority of the proposed hybrid features extraction method, the two-stage feature extraction method [42] was used as comparison. According to the idea, a two-stage feature extraction method (EMD-MNPE-PCA) combining EMD, MNPE and principle component analysis (PCA) was used. After 13-dimensional MNPE features were obtained using EMD and MNPE extraction procedures, PCA was used as dimension reduction tool to eliminate the correlations between multi-dimensional features. By applying PCA to the 13-dimensional MNPE features, the eigenvalues can be obtained, as shown in Figure 12. It can be seen that the eigenvalues that are left to the fourth eigenvalue can be neglected. Therefore, the first four principle components were integrated into a feature vector.

Another commonly used method is empirical mode decomposition entropy (EMDE) [43].

**Figure 13:**
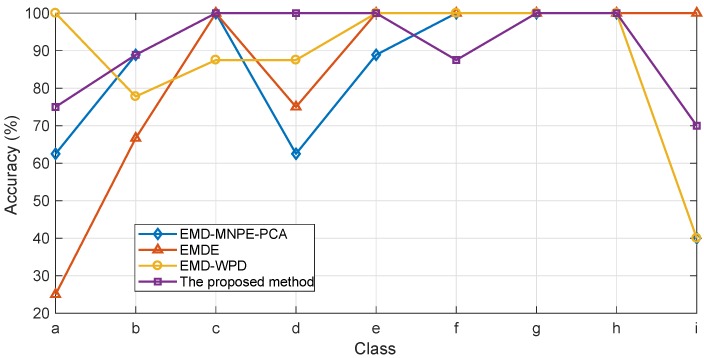
The identification accuracy of each class via IPSO-MSVM using different feature extraction methods.

Finally, the effectiveness of the obtained features was verified via IPSO-MSVM. The identification accuracy of each class and the identification results are given in Figure 13 and Table 3, respectively.

It can be concluded from the results in Table 3 that the overall identification accuracy using the proposed hybrid feature extraction method is the highest. It can also be seen that the proposed hybrid feature extraction method performs better for most classes. Only for Type-f and Type-i it is outperformed by EMDE. Although the identification accuracy using EMDE on Type-i is much higher than the proposed method, it is very low for Type-a and Type-b, which is the result of great similarity between Type-a and Type-b (see Figure 2). EMD-MNPE-PCA only performs a little better than the proposed method on Type-f, but over performs poorly, especially on Type-a, Type-d and Type-i. The previously proposed EMD-WPD performs well for the first eight types of faults, but very poor on the newly added Type-i. Note that, overall, the proposed hybrid feature extraction method performs best.

### 5.4. Discussions

The ultimate aim of this paper is to propose an automatic efficient fault diagnosis method for train plug doors, which is helpful to maintenance staff. Considering the developed signal processing technology and easy-to-collect attributes of sound signals, a sound based fault diagnosis method is proposed. The main contributions of this paper can be summarized as follows.

(1) This paper proposes a novel fault diagnosis method for train plug doors using audio sensors. It can provide a new direction, which may open a new window for fault diagnosis on train plug doors or even more research.

(2) A hybrid feature extraction method combining EMD, MNPE, and the Fisher discrimination criterion is proposed. It can extract the most significant features, which is a guarantee for high recognition accuracy. The optimal scale factor is determined by discussing the relationship between identification accuracy and scale factor.

(3) IPSO-MSVM is proposed and verified using the collected real sound signals with good performance. By comparing with three commonly used classifiers, it performs better on fault diagnosis. It can provide support for maintenance of train plug doors.

However, some limitations also need to be addressed. Although the hybrid feature extraction method combining EMD, MNPE, and the Fisher discrimination criterion can effectively extract fault features, it only considers the first 13 IMFs, whereas the remaining IMFs are ignored, which may contain useful fault information that helps further improve fault identification accuracy.

## 6. Conclusions

At present, the existing fault diagnosis methods for train plug doors mainly belong to post statistical analysis or require much expert experience, which are not very convenient for the maintenance of train plug doors. Aiming at these shortcomings, this paper proposes a novel fault diagnosis method for train plug doors using audio sensors. An efficient hybrid feature extraction method is proposed. First, EMD is used to decompose the sound signals for stationary processing. Then, MNPE features of the first 13 IMFs are extracted. Further, the significant features are obtained using the Fisher discrimination criterion. In addition, the relation between fault identification accuracy and scale factor range is analyzed to determine the optimal scale factor range. The proposed hybrid feature extraction method and IPSO-MSVM were compared to some commonly used methods. Experimental results indicate the proposed method performs well for all classes. Meanwhile, it has the highest identification accuracy of 90.54%, demonstrating its feasibility for fault diagnosis on train plug doors. Besides, the proposed method can provide a way for fault diagnosis of train plug doors, and broaden relevant research fields.

In the future, we will collect more fault types of train plug doors to enrich the fault database. As this work only considers the first 13 IMFs, some available information included in the remaining IMFs may be neglected. Hence, more efficient methods for features extraction need to be developed to improve the identification accuracy.

## Figures and Tables

**Table 1:** Fisher discrimination function values of MNPE features of $imf_{1}$–$imf_{13}$.

	${\mathrm{imf}}_{1}$	${\mathrm{imf}}_{2}$	${\mathrm{imf}}_{3}$	${\mathrm{imf}}_{4}$	${\mathrm{imf}}_{5}$	${\mathrm{imf}}_{6}$	${\mathrm{imf}}_{7}$	${\mathrm{imf}}_{8}$	${\mathrm{imf}}_{9}$	${\mathrm{imf}}_{10}$	${\mathrm{imf}}_{11}$	${\mathrm{imf}}_{12}$	${\mathrm{imf}}_{13}$
*J*	4.22	3.9	6.44	5.99	6.22	9.97	15.84	9.48	4.1	3.61	4.52	3.63	2.06

**Table 2:** The identification results using different classifiers.

Class of	Number of	Number of Correctly Identified Samples			
**Sound Signals**	**Test Samples**	**BP Neural Network**	**1NN**	**PSO-MSVM**	**IPSO-SVM**
a	8	3	4	4	6
b	9	5	9	8	8
c	8	5	6	8	8
d	8	6	7	8	8
e	9	7	4	9	9
f	8	8	8	8	7
g	8	1	8	8	8
h	6	5	6	6	6
i	10	5	6	6	7
Total	74	40	58	65	67
Accuracy (%)		60.81	78.38	87.84	90.54

**Table 3:** The identification results via IPSO-MSVM using different feature extraction methods.

Class of	Number of	Number of Correctly Identified Samples			
**Sound Signals**	**Test Samples**	**EMD-MNPE-PCA**	**EMDE**	**EMD-WPD**	**The Proposed Method**
a	8	5	2	8	6
b	9	8	6	7	8
c	8	8	8	7	8
d	8	5	6	7	8
e	9	8	9	9	9
f	8	8	8	8	7
g	8	8	8	8	8
h	6	6	6	6	6
i	10	4	10	4	7
Total	74	60	63	64	67
Accuracy (%)		81.08	85.14	86.49	90.54

## Abbreviations

The following abbreviations are used in this manuscript:

MNPE	Multi-scale normalized permutation entropy
IPSO	Improved particle swarm optimization
MSVM	Multi-class support vector machine
EMD	Empirical mode decomposition
IMF	Intrinsic mode functions
TBM	Time based maintenance
FTA	Fault tree analysis
FEMA	Failure mode and effects analysis
WPD	Wavelet package decomposition
ANN	Artificial neural network
BP	Backpropagation
CRH5A	China railway CRH5 size A
PCA	Principle component analysis
EMDE	Empirical mode decomposition entropy
